# Regulatory T Cells Prevent Th2 Immune Responses and Pulmonary Eosinophilia during Respiratory Syncytial Virus Infection in Mice

**DOI:** 10.1128/JVI.01295-13

**Published:** 2013-10

**Authors:** Lydia R. Durant, Spyridon Makris, Cornelia Maaike Voorburg, Jens Loebbermann, Cecilia Johansson, Peter J. M. Openshaw

**Affiliations:** Respiratory Infections Section, Department of Respiratory Medicine, National Heart and Lung Institute, Imperial College, London, United Kingdom

## Abstract

During viral infection, inflammation and recovery are tightly controlled by competing proinflammatory and regulatory immune pathways. Respiratory syncytial virus (RSV) is the leading global cause of infantile bronchiolitis, which is associated with recurrent wheeze and asthma diagnosis in later life. Th2-driven disease has been well described under some conditions for RSV-infected mice. In the present studies, we used the *Foxp3*^DTR^ mice (which allow specific conditional depletion of Foxp3^+^ T cells) to investigate the functional effects of regulatory T cells (Tregs) during A2-strain RSV infection. Infected Treg-depleted mice lost significantly more weight than wild-type mice, indicating enhanced disease. This enhancement was characterized by increased cellularity in the bronchoalveolar lavage (BAL) fluid and notable lung eosinophilia not seen in control mice. This was accompanied by abundant CD4^+^ and CD8^+^ T cells exhibiting an activated phenotype and induction of interleukin 13 (IL-13)- and GATA3-expressing Th2-type CD4^+^ T cells that remained present in the airways even 14 days after infection. Therefore, Treg cells perform vital anti-inflammatory functions during RSV infection, suppressing pathogenic T cell responses and inhibiting lung eosinophilia. These findings provide additional evidence that dysregulation of normal immune responses to viral infection may contribute to severe RSV disease.

## INTRODUCTION

Viral lung infections lead to impairment of lung function by multiple mechanisms, including the obstruction of airflow by inflammatory cells in the conducting airways, immune infiltration of lung tissue, and alveolar edema (1, 2). The safe elimination of infection requires a precise control of inflammation, sufficient to control the pathogen load but limiting the severity of immunopathology. An improved understanding of the checks and balances governing the immune response to infection may assist not only in understanding the immune system but also in developing new treatments and in creating effective vaccines (3).

Human respiratory syncytial virus (RSV) infection is the leading cause of severe lower respiratory tract disease, causing infantile bronchiolitis and significant morbidity and mortality in the elderly (1, 4–6). It is estimated that RSV infects 33.8 million children under 5 years old each year, killing between 66,000 and 199,000 children globally. Virtually all these deaths occur in developing countries (7, 8); almost all children have been infected at least once by the age of 3 (8). An overexuberant immune response to infection is thought to underlie the pathogenesis of bronchiolitis. Prevention of RSV infection during infancy reduces the likelihood of recurrent wheezing in later life (9), showing that RSV infection can lead to the development of asthma in some children.

Regulatory T (Treg) cells are a subset of CD4^+^ T cells that specifically express the forkhead box P3 (Foxp3) transcription factor and play an essential role in dampening immune responses and preventing autoimmune disease (10–12). The importance of Treg cells to immune homeostasis is illustrated by humans suffering from immunodysregulation, polyendocrinopathy, enteropathy, X-linked (IPEX) syndrome due to mutations in the *Foxp3* gene (13). This pathology is mimicked in *Foxp3*-deficient *Scurfy* mice, which likewise suffer from a progressive and fatal spontaneous multiorgan inflammatory disease (14, 15).

Treg cells can regulate the response to several viral infections (16–23). During RSV infection, Treg cells limit antigen-specific T cell responses, suppress inflammation, and may help to control viral replication (24–26). However, there is still uncertainty about how Treg cells control the immune response during RSV, in part due to the use of different strategies to deplete Treg cells *in vivo*. Several initial reports used the anti-CD25 PC61 antibody, which binds and depletes cells expressing the high-affinity interleukin 2 (IL-2) receptor alpha chain CD25 (24–26). While CD25 is highly expressed on Foxp3^+^ Treg cells, this receptor is also expressed on activated T cells, and therefore use of PC61 may additionally deplete non-Treg cells (27). More specific depletion strategies have been developed using bacterial artificial chromosome (BAC) transgenic and knock-in mice in which the *Foxp3* gene locus is modified by insertion of a human diphtheria toxin receptor (DTR)-enhanced green fluorescent protein (eGFP) element (28, 29). Upon treatment with diphtheria toxin, the DTR-expressing Foxp3^+^ Treg cells may be specifically killed while all other host cells are unaffected (30). Using BAC-transgenic DEREG (depletion of regulatory T cell) mice, we have previously shown that Tregs require granzyme B expression to cause functional regulation during RSV disease (31). We also have shown that boosting Treg cells using the IL-2/IL-2 receptor (IL-2R) complex ameliorates aspects of disease during primary RSV infection (31) and that RSV disease augmented by prior vaccination with formalin-inactivated vaccine (FI-RSV) is accompanied by the virtual disappearance of Tregs from the airways (32). Importantly, recruitment of Treg cells into the airways by administration of CCL17 and CCL22 intranasally ameliorates vaccine-enhanced FI-RSV disease (32). Taken together, these studies suggest that Tregs modulate the immune response to RSV infection and that severe RSV disease may be caused by dysregulated immune responses to infection.

Previous studies used methods that partially or transiently deplete Tregs. In the case of the DEREG mice, diphtheria toxin administration causes 95 to 98% depletion of Treg cells by apoptosis, but this is transient due to the leaky nature of the BAC transgene; over time, Foxp3^+^ Treg cells that do not express the transgene and thus cannot be depleted appear (30). Therefore, to examine the effects of more-complete Treg cell depletion during RSV infection, we used the *Foxp3*^DTR^ mouse model. The *Foxp*3^DTR^ mice are a knock-in strain created using a targeting vector consisting of a human DTR fused to GFP and containing an internal ribosome entry sequence (IRES) that inserts into the 3′ untranslated region of the *Foxp3* gene locus. Similar to the case with DEREG mice, both the DTR and GFP are exclusively expressed in Foxp3^+^ Treg cells in *Foxp3*^DTR^ mice, and diphtheria toxin treatment in these mice specifically induces apoptosis of Foxp3^+^ Treg cells (33).

Interestingly, although normal C57BL/6 mice are relatively resistant to RSV infection, depletion of Tregs from the C57BL/6-background *Foxp3*^DTR^ mice caused marked weight loss and enhanced cellular influx into the lungs and airways without affecting viral clearance. Inflammatory T cell responses to RSV infection were enhanced, with increased expression of GzmB and CD11a on CD4^+^ and CD8^+^ T cells. Most notably, there was a marked and persistent eosinophilic response in the airways of the Treg-depleted mice, indicative of Th2-mediated pathology. This was associated with increased IL-13^+^ and gamma interferon-positive (IFN-γ^+^) CD4^+^ T cell recruitment to the lungs and enhanced expression of the Th2-defining transcription factor Gata3 in the airways. Thus, we highlight novel functions for Treg cells in shaping the CD4^+^ effector cell response during RSV infection and promoting resolution of pathology.

## MATERIALS AND METHODS

### Mice, virus stocks, and infection.

Six- to ten-week-old C57BL/6 wild-type (purchased from Harlan, United Kingdom) and *Foxp3*^DTR^ mice were housed under specific-pathogen-free conditions according to UK Home Office guidelines.

Plaque-purified human RSV (A2 strain from ATCC) was grown to a high titer in HEp-2 cells. Age-matched wild-type (WT) and Foxp3^DTR^ mice were lightly anesthetized with isofluorane and challenged intranasally (i.n.) with a dose of 8 × 10^5^ focus-forming units (FFU) of RSV on day 0.

### Diphtheria toxin treatment.

*Foxp3*^DTR^ mice were injected with 0.75 μg of diphtheria toxin (DT) (unnicked, Corynebacterium diphtheria from Merck Millipore, United Kingdom) in phosphate-buffered saline (PBS) intraperitoneally (i.p.) on days −1 and 3 during RSV infection to acutely deplete Foxp3^+^ Treg cells.

### Cell isolation and processing.

Mice were culled using a fatal dose (100 to 150 μl) of pentobarbital injected i.p. according the UK Home Office guidelines. Bronchoalveolar lavage (BAL) cells were collected by inserting a syringe with a cannulated needle into the trachea and flushing 3 times with 1 ml of PBS supplemented with 12 mM lidocaine powder (Sigma). Lung lobes were collected and digested with collagenase XI (25 μg/ml; Sigma) using a gentleMACs cell dissociator (Miltenyi Biotech) according to the manufacturer's protocol. Spleen and lung cells were mashed through 100 μM cell strainers to create single-cell suspensions. Total cell counts were determined by flow cytometry using Count Bright counting beads (Invitrogen), and dead cells were excluded by staining for 7-amino-actinomycin D (7-AAD) (Sigma). To determine the cellular composition in the BAL fluid, cells were transferred onto a microscope slide (Thermo Scientific, United Kingdom) using a Shandon Cytospin 3 centrifuge, and slides were stained with hematoxylin and eosin (H&E) (Reagena, Gamidor, United Kingdom). Cells were categorized as macrophages or monocytes, lymphocytes, neutrophils, and eosinophils based on their morphology and size using the Axio Scope.A1 light microscope (Zeiss). Photographs of slides were taken using the AxioCam Erc 5s (Zeiss) at magnification ×40.

### Real-time quantitative PCR for viral load.

Total RNA was extracted from homogenized lung tissue using the Stat60-RNA extraction reagent (AMS Biotechnology Ltd., United Kingdom) and transcribed to cDNA using random hexamers and the Omniscript reverse transcriptase kit (Qiagen, United Kingdom). Real-time quantitative PCR was performed for the RSV large (L) polymerase gene using primers and probes described previously (26) and Quantitect probe PCR master mix (Qiagen). RSV L-gene copy numbers were normalized to the 18S rRNA housekeeping gene.

### Chemokine and cytokine detection.

Eotaxin and IL-5 in the BAL fluid were measured using a custom-made 10-plex Milliplex MAP mouse cytokine/chemokine kit and assayed according to the manufacturer's instructions (Merck Millipore), and data were acquired on the Bio-Plex 200 system (Bio-Rad Laboratories, Hemel Hempstead, United Kingdom). The concentrations of cytokines and chemokines were determined from a standard curve using the Bio-Plex 6 software (Bio-Rad Laboratories). Detection limits were 3.2 pg/ml (lower) and 2,000 pg/ml (upper) for IL-5 and 3.2 pg/ml (lower) and 10,000 pg/ml (upper) for Eotaxin.

### Flow cytometry.

For flow cytometry analysis, dead cells were discriminated using Live/Dead fixable red dead cell stain (Invitrogen) according to the manufacturer's instructions. Cells were incubated in fluorescence-activated cell sorter (FACS) buffer (1× PBS containing 1% bovine serum albumin [BSA] and 5 mM EDTA) with antibody recognizing the Fcγ II/III receptor (BD Biosciences) and then with the following antibodies (purchased from BD) unless otherwise stated: phycoerythrin (PE)-Cy7-conjugated anti-CD3 (clone 145-2C11), V450-conjugated NKp46(CD335) (clone 29A1.4), allophycocyanin (APC) H7- or APC Cy7-conjugated anti-CD4 (clone GK1.5), Alexa Fluor 700-conjugated anti-CD8a (clone 53-6.7), and PE conjugated anti-CD11a (clone 2D7). Intracellular staining of Foxp3, T-bet, Gata3, and GzmB was performed using the Foxp3 staining kit (eBioscience) according to the kit protocol and the following antibodies: Alexa Fluor 488-conjugated anti-Foxp3 (clone FJK-16S), PE-conjugated anti-human/mouse T-bet (clone 4B10), and eFluor 660-conjugated anti-human/mouse Gata3 (clone TWAJ) (all purchased from eBioscience) and APC-conjugated anti-human GzmB (GB12; Invitrogen). Briefly, cells were fixed for 30 min in fixation buffer, washed with FACS buffer, and then incubated in permeabilization buffer with antibodies for 1 h.

For intracellular detection of IFN-γ and IL-13, fresh cells were stimulated in 96-well plates with 100 ng/ml of phorbol myristate acetate (PMA) and 1 μg/ml of ionomycin or with 5 μg/ml of M peptide (187-195; NAITNAKII; Anaspec) in complete Dulbecco modified Eagle medium (DMEM) supplemented with 10% fetal bovine serum, 2 mM l-glutamine, 100 U/ml penicillin, and 100 μg/ml streptomycin. After 1 h of incubation, monensin (Golgi Stop; BD) was added. Cells were incubated for a further 3 h, washed, and stained for surface receptors. Intracellular cytokine staining was performed using the Cytofix/Cytoperm kit (BD) according to the manufacturer's instructions using peridinin chlorophyll protein (PerCP) Cy5.5-conjugated anti-IFN-γ antibody (clone XMG1.2; eBioscience) and PE-conjugated anti-IL-13 antibody (clone eBio13a; eBioscience). Stimulation with the M peptide was for 6 h, and monensin was added after 1 h. Cells were acquired on the LSR II or the Fortessa (BD) instrument, and data were analyzed using FlowJo software (version 7.6.5). Cells were gated for live cells, singlets, and lymphocytes and then analyzed for the relevant markers.

### Statistical analysis.

Results are presented as means ± standard errors of the means (SEM). Statistical significance was determined using a nonparametric Mann-Whitney test (∗, *P* ≤ 0.05; ∗∗, *P* ≤ 0.01; ∗∗∗, *P* ≤ 0.001; ∗∗∗∗, *P* < 0.0001). *P* values of <0.05 were considered significant (Prism software; Graph-Pad Software Inc.).

## RESULTS

### Regulatory T cells limit and control inflammation after RSV infection.

C57BL/6 mice can be infected with RSV but are more resistant to RSV-induced illness than the BALB/c mice used in most RSV studies (31). We infected wild-type (WT) C57BL/6 or *Foxp3*^DTR^ (DTR/DT) C57BL/6 mice intranasally with 8 × 10^5^ FFU of human A2 RSV on day 0. *Foxp3*^DTR^ (DTR/DT) mice were depleted of Foxp3^+^ Treg cells by injection of diphtheria toxin (DT) intraperitoneally (i.p.) on days −1 and 3. Depletion was highly effective, removing >90% of Treg cells from the lungs of *Foxp3*^DTR^ mice (DTR/DT/RSV) on day 4 postinfection compared to results for wild-type (WT/RSV) controls (see Fig. S1A in the supplemental material). The frequency of Tregs remained significantly reduced to day 8 in both the airways (BAL) and lungs of depleted DTR/DT mice (see Fig. S1B), although the total numbers of Foxp3^+^ Treg cells rebounded by day 8 postinfection in the airways (see Fig. S1C). A significant loss of Treg cells was also seen in uninfected Treg-depleted mice (DTR/DT) compared to results for naive WT controls (see Fig. S1A and B).

**Fig 1:**
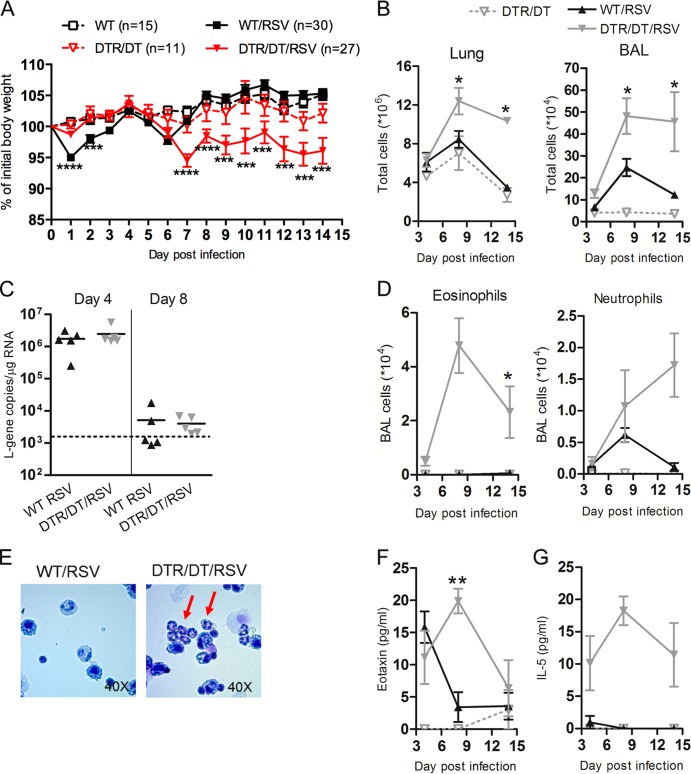
Regulatory T cells attenuate and modify responses to RSV infection. Wild-type (WT/RSV) or Treg-depleted *Foxp3*^DTR^ (DTR/DT/RSV) mice were infected with A2 strain RSV intranasally on day 0. Uninfected wild-type (WT) or Treg-depleted (DTR/DT) mice were used as controls. (A) Illness was monitored by recording individual body weights daily, and the mean (± SEM) weight for each group, measured as a percentage of the day 0 (initial) weight, is shown. Data are pooled from three experiments. (B) Total cells in the lung and bronchoalveolar lavage (BAL) fluid were quantified as a measure of inflammation. (C) Expression of the RSV L gene was quantified in the lung on days 4 and 8 postinfection by quantitative PCR. The dotted line shows the detection limit for the copy numbers of L-gene according to the standard curve. (D) Numbers of eosinophils and neutrophils in the BAL fluid were determined by performing H&E staining on cytospin slides and quantifying numbers by differential cell counts. (E) Representative BAL cytospins from day 14 are shown with red arrows indicating eosinophils in Treg-depleted mice. BAL fluid supernatants were analyzed for eotaxin (F) or IL-5 (G) protein levels using a multiplex kit. Data are representative of two or three independent experiments (*n* = 4 to 5 mice per group) unless otherwise indicated. WT/RSV versus DTR/DT/RSV: ∗∗∗∗, *P* < 0.0001; ∗∗∗, *P* < 0.001; ∗∗, *P* < 0.01; ∗, *P* < 0.05.

Using weight loss as a measure of disease, we found that the depletion of Tregs (DTR/DT/RSV) made mice more susceptible to disease, enhancing weight loss beginning at day 7 and persisting until day 14 postinfection compared to results for wild-type controls (WT/RSV) (Fig. 1A). Pulmonary inflammation (assessed by determining total cellular influx in the lungs and airways [BAL fluid]) was significantly enhanced on day 8 postinfection in Treg-depleted DTR/DT/RSV mice compared to that of WT/RSV controls (Fig. 1B). Furthermore, the inflammation persisted until at least day 14 postinfection in Treg-depleted mice.

One explanation for the enhanced pathology observed in the absence of Treg cells could be an inability to clear the virus. However, when the RSV L gene was measured in the lungs of mice on day 4 (peak virus load) and day 8 (virus clearance) postinfection, there was no detectable difference between WT and Treg-depleted mice (Fig. 1C). Instead, H&E staining of cytospins of the airway (BAL) cells showed a greater influx of eosinophils in Treg-depleted DTR/DT/RSV mice but not in WT/RSV control mice (Fig. 1D). Both eosinophils and neutrophils were detected in Treg-depleted mice until day 14 postinfection (Fig. 1D and E), indicative of an immune-mediated inflammation. Furthermore, this influx was associated with an increase in eotaxin levels in the BAL fluid of Treg-depleted mice compared to findings for WT controls (Fig. 1F). Additionally, the Th2-associated cytokine interleukin 5 (IL-5) was detectable only in the airways of Treg-depleted mice and not in those of WT mice following RSV infection (Fig. 1G). Together, these findings suggest that Treg cells are not essential for viral clearance but instead control the intensity and type of inflammatory response in the face of viral infection.

### T cell responses are enhanced in the absence of Tregs.

**Fig 2:**
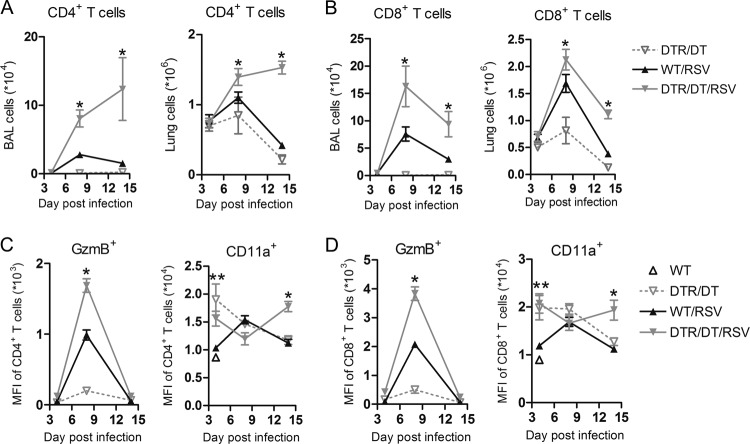
Treg depletion leads to enhanced activation and recruitment of T cells into the lungs and airways during RSV infection. Following infection of wild-type (WT/RSV) and Treg-depleted (DTR/DT/RSV) mice with RSV, whole lung and bronchoalveolar lavage (BAL) fluid were harvested on days 4, 8, and 14 postinfection. CD4^+^ (A) or CD8^+^ (B) T cells were quantified in the BAL fluid and lung by flow cytometry analysis. Expression (mean fluorescence intensity [MFI]) of activation markers, including GzmB and CD11a, on both CD4^+^ (C) and CD8^+^ (D) T cells in the lung are shown. Data are representative of two independent experiments (*n* = 4 to 5 mice per group). WT/RSV versus DTR/DT/RSV: ∗, *P* ≤ 0.05; ∗∗, *P* ≤ 0.01.

Loss of Treg cells resulted in enhanced weight loss, peaking at day 7 (Fig. 1A), which coincides with an exuberant adaptive immune response. Therefore, we further characterized the T lymphocyte response in the lung and airways using flow cytometry. While there was a notable influx of both CD4^+^ and CD8^+^ T cells in the lungs and airways of WT mice infected with RSV compared to results for uninfected (DTR/DT) controls, both T cell subsets were significantly increased on day 8 postinfection in the lungs and airways of Treg-depleted (DTR/DT/RSV) mice, and T cell numbers remained heightened to day 14 postinfection (Fig. 2A and B). We further examined T cell activation by measuring expression of granzyme B (GzmB), a marker of cytolytic activity, and CD11a (integrin alpha L), a component of LFA-1 and a marker associated with antiviral T cell responses (34). The mean fluorescence intensity (MFI) of GzmB but not CD11a was increased on both CD4^+^ and CD8^+^ T cells in the lungs of WT mice infected with RSV compared to results for uninfected (DTR/DT) controls (Fig. 2C and D). However, expression of both activation markers was even further enhanced on CD4^+^ and CD8^+^ T cells in the lungs of Treg-depleted mice infected with RSV, and this was significant at day 8 (for GzmB) or day 14 (for CD11a) postinfection. Therefore, these data support that overactive and persistent effector T cell responses contribute to immune pathology in Treg-depleted mice during RSV infection.

### Antigen-specific CD8^+^ T cells are increased in the respiratory tract and spleen of Treg-depleted mice following RSV challenge.

Upon primary RSV challenge, there is a rapid expansion of antigen-specific CD8^+^ T cells that contribute to effective viral clearance but may also lead to tissue pathology. The predominant CD8^+^ T cell epitope induced by RSV challenge in C57BL/6 mice recognizes the RSV M protein (34). Therefore, we quantified the M-peptide-specific CD8^+^ T cell response in order to determine if increased antigen-specific T cell responses could offer an explanation for the enhanced pathology with loss of Treg cells.

**Fig 3:**
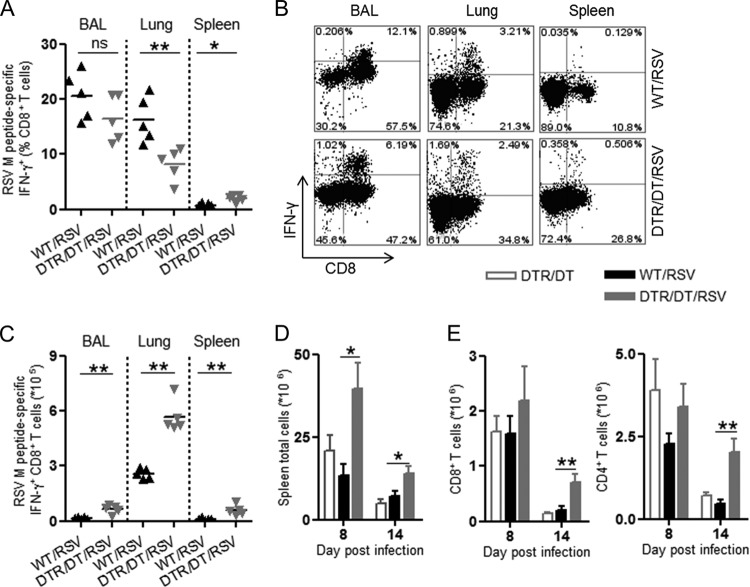
Peptide-specific CD8^+^ T cell responses are enhanced in the respiratory tract and spleens of Treg-depleted mice during RSV infection. Tissues were harvested from wild-type (WT/RSV) or Treg-depleted (DTR/DT/RSV) mice infected with RSV at various days postinfection. Following a 6-h restimulation with the RSV M peptide, IFN-γ^+^ CD8^+^ T cells were quantified in the BAL fluid, lung, and spleen by FACS. (A and B) The frequencies of CD8^+^ T cells expressing M-peptide-specific IFN-γ among total CD8^+^ T cells on day 8 postinfection are shown (A), with representative FACs plots (B) for the various tissues examined. (C) Total numbers of M-peptide-specific IFN-γ^+^ CD8^+^ T cells in all the tissues from Treg-depleted or WT mice on day 8 postinfection. Spleens were harvested from RSV-infected WT or Treg-depleted mice on days 8 and 14 postinfection. (D and E) Total spleen cells were quantified (D), and total numbers of CD8^+^ and CD4^+^ T cells were enumerated (E). Mice depleted of Treg cells (DTR/DT) but not infected with RSV were used as controls. Data are representative of two or three independent experiments. ∗, *P* < 0.05; ∗∗, *P* < 0.01; ns, not significant.

Lungs, BAL fluid, and spleens were isolated, and cell homogenates were stimulated with RSV M_187–195_ peptide for 6 h, following which IFN-γ expression was measured by intracellular flow cytometry. As expected, on day 8 post-infection of WT mice, a clear proportion of CD8^+^ T cells expressed IFN-γ in response to stimulation with M peptide, and the majority of these were localized to the airways (BAL fluid; 16 to 26%) and lungs (11 to 20%) (Fig. 3A and B). There were a similar proportion of M-peptide-specific CD8^+^ T cells in the BAL fluid of control mice and Treg-depleted DTR mice despite the enhanced pathology in the Treg-depleted mice. However, the total numbers of M-peptide-specific CD8^+^ T cells in Treg-depleted mice were increased in all of the tissues examined compared to results for WT mice (Fig. 3C), including results for the spleen, which suggests a less localized antiviral T cell response in the absence of Treg cells.

We found that Treg-depleted RSV-infected mice also develop a notable splenomegaly compared to WT mice, an effect persisting until day 14 postinfection (Fig. 3D). The splenomegaly was characterized by expansion of both CD4^+^ and CD8^+^ T cells, providing evidence of a systemic inflammatory T cell response (Fig. 3E) after infection and Treg depletion (Fig. 3D and E). The weight loss in infected Treg-depleted mice therefore seems to be accounted for by enhanced local recruitment of inflammatory cells to the lung, an excessive RSV-specific CD8^+^ T cell response, and evidence of systemic T cell activation.

### Enhanced Th2-type airway responses in Treg-depleted mice.

**Fig 4:**
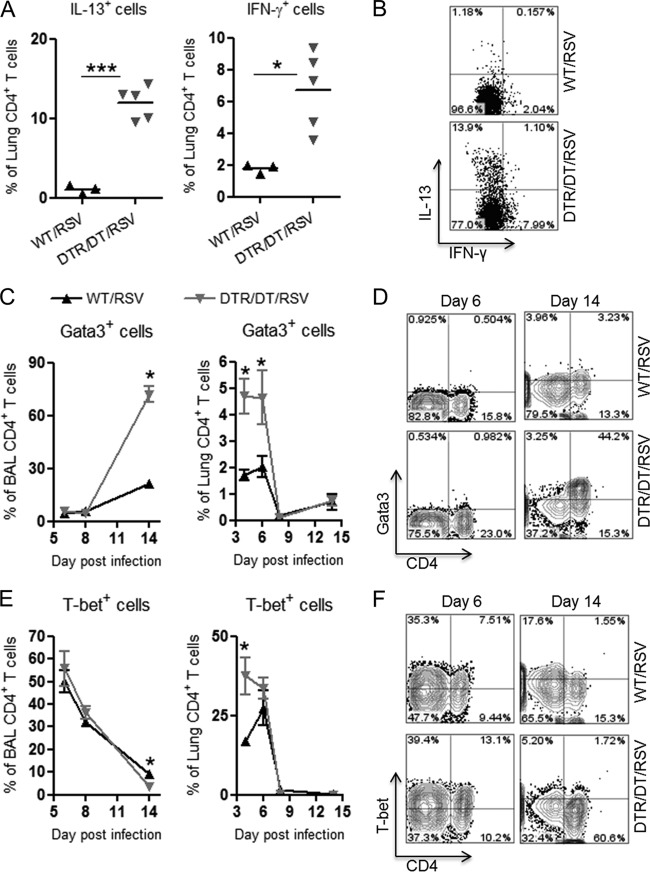
Regulatory T cells limit pathogenic Th2-type responses in the airways following RSV infection. Wild-type (WT/RSV) and Treg-depleted (DTR/DT/RSV) mice were challenged with RSV, and tissues were harvested to examine CD4^+^ T cell responses. (A and B) The frequency of CD4^+^ T cells expressing cytokines IL-13 and IFN-γ were quantified in the lung on day 4 postinfection by flow cytometry (A), and representative FACS plots are shown (B). (C and D) The frequencies of CD4^+^ T cells expressing the transcription factor Gata3 in the BAL fluid and the lung at various days postinfection are shown (C), with representative FACS plots from the BAL fluid (D). (E and F) The frequencies of CD4^+^ T cells expressing the transcription factor T-bet in the BAL fluid and lung at various days postinfection (E) with representative FACS plots from the BAL fluid (F) are shown. Data are representative of two independent experiments (*n* = 3 to 4 mice per group). ∗, *P* < 0.05; ∗∗∗, *P* < 0.001.

In addition to antiviral CD8^+^ T cell responses, other factors might contribute to excessive pulmonary pathology in the Treg-depleted mice, and thus we decided to further characterize the CD4^+^ T cell responses developing in the lung and airways following RSV challenge. To address this, we examined expression of Th1- and Th2-specifying cytokines and transcription factors using intracellular flow cytometry analysis of lung and BAL cells. Interestingly, at an early time point (day 4 postinfection), there was a significant increase in the proportion of CD4^+^ T cells expressing either IL-13 or IFN-γ in the lungs of Treg-depleted mice compared to results for WT controls, indicative of a mixed Th1- and Th2-type response (Fig. 4A and B). Additionally, expression of the Th2-associated transcription factor Gata3 was increased in lung cells on days 4 and 6 and in BAL cells on day 14 (Fig. 4C and D), while the Th1-specifying transcription factor T-bet was enhanced on day 4 in the lungs only (Fig. 4E) in Treg-depleted mice compared to WT controls. By day 14, the majority (70%) of CD4^+^ T cells in the BAL fluid expressed Gata3, while only a small proportion (3%) expressed T-bet in Treg-depleted mice (Fig. 4C to F). These findings correlate with the marked eosinophilia in the airways of Treg-depleted mice (Fig. 1D), also indicative of a Th2-type immune response.

Previous studies have shown that Foxp3^+^ Treg cells may also acquire expression of T helper cell-specifying transcription factors, and this appears to be important for their suppressive function in different inflammatory settings (35–38). Therefore, we examined expression of T-bet and Gata3 on Foxp3^+^ Treg cells in the airways of WT and Treg-depleted mice following RSV infection. In WT mice, there was a clear proportion (10%) of CD4^+^ T cells in the BAL fluid that coexpressed the Treg cell marker Foxp3 and the Th1 cell marker T-bet at the peak of the adaptive response (days 6 and 8 postinfection) (see Fig. S2A in the supplemental material). In the Treg-depleted mice, there were very few Tregs at this time point (see Fig. S2A). However, at day 14 postinfection, Foxp3^+^ Treg cells reappeared in the airways of the Treg-depleted group and a high proportion (25%) of these cells coexpressed the Th2 marker Gata3 (see Fig. S2B). In WT mice, the majority of Foxp3^+^ Treg cells left the airways by day 14, since pathology had resolved, and instead appeared at baseline proportions in the lungs (see Fig. S2C). These findings render further support that Treg cells function to limit effector T cell responses in the airways of RSV-challenged mice and may switch their phenotype according to the type of inflammatory milieu.

Overall, this study provides further evidence for the essential role Treg cells play in controlling inflammatory T cell responses and pathology in RSV infection. In particular, we have highlighted novel roles for Treg cells in promoting resolution of disease by day 14 postinfection and preventing the persistence of Th2-type inflammation and eosinophilia.

## DISCUSSION

Pulmonary disease caused by RSV infection involves a complex interplay of inflammatory and anti-inflammatory immune responses, tightly regulated by Foxp3^+^ Treg cells. We have now provided evidence that Treg cells not only dampen overexuberant and pathological T cell responses but also limit Th2-type immune responses (e.g., Gata3- and IL-13-expressing CD4^+^ T cells and eosinophilic influx into the airways). Additionally, Treg cells suppress antiviral CD8^+^ T cell responses and prevent systemic manifestations in RSV infection. Our findings therefore support the concept that Tregs play a central role in preventing pathology in viral bronchiolitis.

Perhaps the most exciting finding from our studies was that acute Treg cell depletion in the *Foxp3*^DTR^ mice led to the appearance of a phased and mixed Th1/Th2-type immune response following primary RSV infection. Previous studies have shown that the acute loss of Treg cells alone can lead to a Th2-type immune response and pathology without any additional antigenic stimulation (39), and in allergic inflammation, acute Treg depletion at the time of sensitization (but not challenge) leads to an enhanced Th2-type immune response and worsening of pathology (40–43). Mixed Th1/Th2-type immune responses (including eosinophils, neutrophils, and both IL-13- and IFN-γ-producing CD4^+^ T cells) are also a feature of vaccine-enhanced RSV disease in mice and of human bronchiolitis (44–46), indicating that dysregulation is the hallmark of RSV disease.

We showed previously that RSV-infected Treg-depleted DEREG mice (as well as mice infected with RSV as neonates and rechallenged with RSV as adults) have marked eosinophilia associated with increased disease severity (31, 47). Importantly, in the Treg-depleted *Foxp3*^DTR^ mice, both the eosinophils and Th2 cells persist beyond the peak of the antiviral immune response and viral clearance, suggesting that they directly contribute to the persistent pathology as reflected by delayed recovery from weight loss. Thus, Treg cells not only drive an appropriate antiviral response during RSV infection but also promote resolution of the disease.

Even more interesting was the observation that Foxp3^+^ Treg cells in the airways acquired expression of Gata3, a marker of Th2 cells, in *Foxp3*^DTR^ mice at later time points (day 14) following RSV infection. Previous studies have reported expression of Gata3 by Foxp3^+^ Treg cells in mucosal sites like the intestine and skin and have shown that expression of Gata3 is important for Treg cell-mediated suppression of inflammation at these sites (38). In the FI-RSV vaccine model, trafficking of Treg cells to the airways is dependent on CCL17 and CCL22, chemokines typically associated with Th2 cell responses and the influx of Treg cells is important in resolution of Th2-type inflammation (32). Similarly, in the ovalbumin (OVA) model of allergic inflammation, expression of CCR4 (the receptor for CCL17 and CCL22) is important in the ability of Tregs to suppress Th2 pathology (43). However, mice in which tolerance to maternal OVA was induced during their weaning period and which were then infected with RSV displayed an excessive Th2-type pathology, and this included expression of both Gata3 and Th2-type cytokines by Foxp3^+^ Treg cells, suggesting that these cells acquire effector functions and contribute to worsened disease (48). Therefore, it would be interesting in future studies to evaluate the role of these Gata3-expressing Treg cells in RSV disease and to determine whether they maintain their suppressive function or are converting to effector T cells that contribute to pathology.

Recent work has shown that induced Treg cells (iTregs) (those generated in the tissues) rather than thymically derived natural Treg cells (nTregs) are important for controlling Th2 responses and that specific depletion of iTreg cells by mutating a conserved noncoding region in the *Foxp3* gene (*Foxp3*^ΔCNS1^) resulted in pathology (49). Although we did not distinguish between natural and induced Treg cells in our model, it would be interesting in further studies to determine whether RSV infection in the *Foxp3*^ΔCNS1^ mouse model results in a similar enhanced pathology or whether natural Tregs are capable of controlling the immune response.

In RSV infection, there are conflicting data concerning the role of Treg cells in regulating the influx of antigen-specific CD8^+^ T cells in the lungs, and previous studies demonstrate that depletion of Treg cells can either in result an inhibited or enhanced antigen-specific T cell response (24–26, 31). We observed an enhanced number of peptide-specific CD8^+^ T cells in the absence of Treg cells in all the tissues, including the spleen, after RSV infection. This would suggest that Treg cells are important for controlling virus-specific T cell responses not just in the airways but also systemically. Furthermore, the excessive inflammatory milieu that develops in the Treg-depleted mice could directly contribute to aberrant activation and homing of T effector cells to a variety of tissue sites outside the respiratory tract, thus prolonging disease resolution. In fact, previous work has indicated that CD8^+^ T cells may be critical for controlling excessive Th2 responses and eosinophilia in vaccine-enhanced RSV disease models (50, 51). Therefore, the loss of CD8^+^ T cells from the airways by day 14 postinfection, while the eosinophils and CD4^+^ Th2 cells persisted, may further indicate a lack of control of these pathological immune cells and contribute to the persistent disease in the respiratory tract.

Despite the enhanced antiviral T cell response, we did not see any difference in viral loads in the lungs of WT and Treg-depleted mice, and these findings contrast with those of previous studies in our group using the DEREG model of acute Treg cell depletion (31). Similar to the *Foxp3*^DTR^ model, DT treatment of DEREG mice is effective at short-term systemic depletion of Foxp3^+^ Treg cells; however, DEREG mice will not develop systemic autoimmunity due to the gradual outgrowth of a suppressive Foxp3^+^ GFP^−^ population (28). In contrast, *Foxp3*^DTR^ adult mice will succumb to a fatal autoimmunity as early as 10 days after the first DT treatment if treatments are given every other day (33). While experiments in this study involved only a short DT treatment course to avoid complications from the systemic disease, it is certainly possible that the enhanced inflammation that develops in the *Foxp3*^DTR^ mice could explain differences in antigen-specific responses, pathological outcome, and viral clearance compared to findings of previous studies using the DEREG mice or anti-CD25 treatment.

In conclusion, these studies provide further support for the concept that Tregs perform a vital function in controlling the immune response during primary RSV infections. With inadequate Treg responses, lung pathology is enhanced and prolonged, with an immune phenotype similar to that seen in asthma. Although these effects may be transient and present only in certain phases of disease, the long-term consequences of Th2 “imprinting” of the respiratory mucosa (52) may account for the association between severe infantile RSV infection and the development of wheezing disorders in later childhood (9). If this is the case, strategies that boost Treg responses might not only ameliorate disease but also result in long-term improvements in respiratory health.

## Supplementary Material

Supplemental material

## References

[1] Openshaw PJ 2005 Antiviral immune responses and lung inflammation after respiratory syncytial virus infection. Proc. Am. Thorac. Soc. 2:121–125. 16113479 10.1513/pats.200504-032AW

[2] Habibi MS Openshaw PJ 2012 Benefit and harm from immunity to respiratory syncytial virus: implications for treatment. Curr. Opin. Infect. Dis. 25:687–694. 23086186 10.1097/QCO.0b013e32835a1d92

[3] Openshaw PJ Chiu C 2013 Protective and dysregulated T cell immunity in RSV infection. Curr. Opin. Virol. 10.1016/j.coviro.2013.05.005. PMC4295022 23806514

[4] Openshaw PJ Dean GS Culley FJ 2003 Links between respiratory syncytial virus bronchiolitis and childhood asthma: clinical and research approaches. Pediatr. Infect. Dis. J. 22:S58–S64. 12671454 10.1097/01.inf.0000053887.26571.eb

[5] Collins PL Graham BS 2008 Viral and host factors in human respiratory syncytial virus pathogenesis. J. Virol. 82:2040–2055. 17928346 10.1128/JVI.01625-07 PMC2258918

[6] Hall CB Weinberg GA Iwane MK Blumkin AK Edwards KM Staat MA Auinger P Griffin MR Poehling KA Erdman D Grijalva CG Zhu Y Szilagyi P 2009 The burden of respiratory syncytial virus infection in young children. N. Engl. J. Med. 360:588–598. 19196675 10.1056/NEJMoa0804877 PMC4829966

[7] Graham BS 2011 Biological challenges and technological opportunities for respiratory syncytial virus vaccine development. Immunol. Rev. 239:149–166. 21198670 10.1111/j.1600-065X.2010.00972.x PMC3023887

[8] Nair H Nokes DJ Gessner BD Dherani M Madhi SA Singleton RJ O'Brien KL Roca A Wright PF Bruce N Chandran A Theodoratou E Sutanto A Sedyaningsih ER Ngama M Munywoki PK Kartasasmita C Simões EA Rudan I Weber MW Campbell H 2010 Global burden of acute lower respiratory infections due to respiratory syncytial virus in young children: a systematic review and meta-analysis. Lancet 375:1545–1555. 20399493 10.1016/S0140-6736(10)60206-1 PMC2864404

[9] Blanken MO Rovers MM Molenaar JM Winkler-Seinstra PL Meijer A Kimpen JL Bont L 2013 Respiratory syncytial virus and recurrent wheeze in healthy preterm infants. N. Engl. J. Med. 368:1791–1799. 23656644 10.1056/NEJMoa1211917

[10] Fontenot JD Gavin MA Rudensky AY 2003 Foxp3 programs the development and function of CD4+CD25+ regulatory T cells. Nat. Immunol. 4:330–336. 12612578 10.1038/ni904

[11] Josefowicz SZ Lu LF Rudensky AY 2012 Regulatory T cells: mechanisms of differentiation and function. Annu. Rev. Immunol. 30:531–564. 22224781 10.1146/annurev.immunol.25.022106.141623 PMC6066374

[12] Hori S Nomura T Sakaguchi S 2003 Control of regulatory T cell development by the transcription factor Foxp3. Science 299:1057–1061. 12522256 10.1126/science.1079490

[13] Bennett CL Christie J Ramsdell F Brunkow ME Ferguson PJ Whitesell L Kelly TE Saulsbury FT Chance PF Ochs HD 2001 The immune dysregulation, polyendocrinopathy, enteropathy, X-linked syndrome (IPEX) is caused by mutations of FOXP3. Nat. Genet. 27:20–21. 11137993 10.1038/83713

[14] Wildin RS Ramsdell F Peake J Faravelli F Casanova JL Buist N Levy-Lahad E Mazzella M Goulet O Perroni L Bricarelli FD Byrne G McEuen M Proll S Appleby M Brunkow ME 2001 X-linked neonatal diabetes mellitus, enteropathy and endocrinopathy syndrome is the human equivalent of mouse scurfy. Nat. Genet. 27:18–20. 11137992 10.1038/83707

[15] Brunkow ME Jeffery EW Hjerrild KA Paeper B Clark LB Yasayko SA Wilkinson JE Galas D Ziegler SF Ramsdell F 2001 Disruption of a new forkhead/winged-helix protein, scurfin, results in the fatal lymphoproliferative disorder of the scurfy mouse. Nat. Genet. 27:68–73. 11138001 10.1038/83784

[16] Fernandez MA Puttur FK Wang YM Howden W Alexander SI Jones CA 2008 T regulatory cells contribute to the attenuated primary CD8+ and CD4+ T cell responses to herpes simplex virus type 2 in neonatal mice. J. Immunol. 180:1556–1564. 18209051 10.4049/jimmunol.180.3.1556

[17] Lund JM Hsing L Pham TT Rudensky AY 2008 Coordination of early protective immunity to viral infection by regulatory T cells. Science 320:1220–1224. 18436744 10.1126/science.1155209 PMC2519146

[18] Veiga-Parga T Suryawanshi A Mulik S Gimenez F Sharma S Sparwasser T Rouse BT 2012 On the role of regulatory T cells during viral-induced inflammatory lesions. J. Immunol. 189:5924–5933. 23129753 10.4049/jimmunol.1202322 PMC3518750

[19] Lanteri MC O'Brien KM Purtha WE Cameron MJ Lund JM Owen RE Heitman JW Custer B Hirschkorn DF Tobler LH Kiely N Prince HE Ndhlovu LC Nixon DF Kamel HT Kelvin DJ Busch MP Rudensky AY Diamond MS Norris PJ 2009 Tregs control the development of symptomatic West Nile virus infection in humans and mice. J. Clin. Invest. 119:3266–3277. 19855131 10.1172/JCI39387 PMC2769173

[20] Betts RJ Ho AW Kemeny DM 2011 Partial depletion of natural CD4+ CD25+ regulatory T cells with anti-CD25 antibody does not alter the course of acute influenza A virus infection. PLoS One 6:e27849. 10.1371/journal.pone.0027849. 22125630 PMC3220674

[21] Betts RJ Prabhu N Ho AW Lew FC Hutchinson PE Rotzschke O Macary PA Kemeny DM 2012 Influenza A virus infection results in a robust, antigen-responsive, and widely disseminated Foxp3+ regulatory T cell response. J. Virol. 86:2817–2825. 22205730 10.1128/JVI.05685-11 PMC3302292

[22] Cervantes-Barragan L Firner S Bechmann I Waisman A Lahl K Sparwasser T Thiel V Ludewig B 2012 Regulatory T cells selectively preserve immune privilege of self-antigens during viral central nervous system infection. J. Immunol. 188:3678–3685. 22407917 10.4049/jimmunol.1102422

[23] Keynan Y Card CM McLaren PJ Dawood MR Kasper K Fowke KR 2008 The role of regulatory T cells in chronic and acute viral infections. Clin. Infect. Dis. 46:1046–1052. 18444822 10.1086/529379

[24] Ruckwardt TJ Bonaparte KL Nason MC Graham BS 2009 Regulatory T cells promote early influx of CD8+ T cells in the lungs of respiratory syncytial virus-infected mice and diminish immunodominance disparities. J. Virol. 83:3019–3028. 19153229 10.1128/JVI.00036-09 PMC2655550

[25] Fulton RB Meyerholz DK Varga SM 2010 Foxp3+ CD4 regulatory T cells limit pulmonary immunopathology by modulating the CD8 T cell response during respiratory syncytial virus infection. J. Immunol. 185:2382–2392. 20639494 10.4049/jimmunol.1000423 PMC2923480

[26] Lee DC Harker JA Tregoning JS Atabani SF Johansson C Schwarze J Openshaw PJ 2010 CD25+ natural regulatory T cells are critical in limiting innate and adaptive immunity and resolving disease following respiratory syncytial virus infection. J. Virol. 84:8790–8798. 20573822 10.1128/JVI.00796-10 PMC2919030

[27] Setiady YY Coccia JA Park PU 2010 In vivo depletion of CD4+FOXP3+ Treg cells by the PC61 anti-CD25 monoclonal antibody is mediated by FcgammaRIII+ phagocytes. Eur. J. Immunol. 40:780–786. 20039297 10.1002/eji.200939613

[28] Lahl K Loddenkemper C Drouin C Freyer J Arnason J Eberl G Hamann A Wagner H Huehn J Sparwasser T 2007 Selective depletion of Foxp3+ regulatory T cells induces a scurfy-like disease. J. Exp. Med. 204:57–63. 17200412 10.1084/jem.20061852 PMC2118432

[29] Kim J Lahl K Hori S Loddenkemper C Chaudhry A deRoos P Rudensky A Sparwasser T 2009 Cutting edge: depletion of Foxp3+ cells leads to induction of autoimmunity by specific ablation of regulatory T cells in genetically targeted mice. J. Immunol. 183:7631–7634. 19923467 10.4049/jimmunol.0804308

[30] Lahl K Sparwasser T 2011 In vivo depletion of FoxP3+ Tregs using the DEREG mouse model. Methods Mol. Biol. 707:157–172. 21287334 10.1007/978-1-61737-979-6_10

[31] Loebbermann J Thornton H Durant L Sparwasser T Webster KE Sprent J Culley FJ Johansson C Openshaw PJ 2012 Regulatory T cells expressing granzyme B play a critical role in controlling lung inflammation during acute viral infection. Mucosal Immunol. 5:161–172. 22236998 10.1038/mi.2011.62 PMC3282434

[32] Loebbermann J Durant L Thornton H Johansson C Openshaw PJ 2013 Defective immunoregulation in RSV vaccine-augmented viral lung disease restored by selective chemoattraction of regulatory T cells. Proc. Natl. Acad. Sci. U. S. A. 110:2987–2992. 23382205 10.1073/pnas.1217580110 PMC3581918

[33] Kim JM Rasmussen JP Rudensky AY 2007 Regulatory T cells prevent catastrophic autoimmunity throughout the lifespan of mice. Nat. Immunol. 8:191–197. 17136045 10.1038/ni1428

[34] Lukens MV Claassen EA de Graaff PM van Dijk ME Hoogerhout P Toebes M Schumacher TN van der Most RG Kimpen JL van Bleek GM 2006 Characterization of the CD8+ T cell responses directed against respiratory syncytial virus during primary and secondary infection in C57BL/6 mice. Virology 352:157–168. 16730775 10.1016/j.virol.2006.04.023

[35] Chaudhry A Rudra D Treuting P Samstein RM Liang Y Kas A Rudensky AY 2009 CD4+ regulatory T cells control TH17 responses in a Stat3-dependent manner. Science 326:986–991. 19797626 10.1126/science.1172702 PMC4408196

[36] Zheng Y Chaudhry A Kas A deRoos P Kim JM Chu TT Corcoran L Treuting P Klein U Rudensky AY 2009 Regulatory T-cell suppressor program co-opts transcription factor IRF4 to control T(H)2 responses. Nature 458:351–356. 19182775 10.1038/nature07674 PMC2864791

[37] Koch MA Tucker-Heard G Perdue NR Killebrew JR Urdahl KB Campbell DJ 2009 The transcription factor T-bet controls regulatory T cell homeostasis and function during type 1 inflammation. Nat. Immunol. 10:595–602. 19412181 10.1038/ni.1731 PMC2712126

[38] Wohlfert EA Grainger JR Bouladoux N Konkel JE Oldenhove G Ribeiro CH Hall JA Yagi R Naik S Bhairavabhotla R Paul WE Bosselut R Wei G Zhao K Oukka M Zhu J Belkaid Y 2011 GATA3 controls Foxp3+ regulatory T cell fate during inflammation in mice. J. Clin. Invest. 121:4503–4515. 21965331 10.1172/JCI57456 PMC3204837

[39] Lahl K Mayer CT Bopp T Huehn J Loddenkemper C Eberl G Wirnsberger G Dornmair K Geffers R Schmitt E Buer J Sparwasser T 2009 Nonfunctional regulatory T cells and defective control of Th2 cytokine production in natural scurfy mutant mice. J. Immunol. 183:5662–5672. 19812199 10.4049/jimmunol.0803762

[40] Fyhrquist N Lehtimäki S Lahl K Savinko T Lappeteläinen AM Sparwasser T Wolff H Lauerma A Alenius H 2012 Foxp3+ cells control Th2 responses in a murine model of atopic dermatitis. J. Investig. Dermatol. 132:1672–1680. 22402436 10.1038/jid.2012.40

[41] Baru AM Hartl A Lahl K Krishnaswamy JK Fehrenbach H Yildirim AO Garn H Renz H Behrens GM Sparwasser T 2010 Selective depletion of Foxp3+ Treg during sensitization phase aggravates experimental allergic airway inflammation. Eur. J. Immunol. 40:2259–2266. 20544727 10.1002/eji.200939972

[42] Baru AM Ganesh V Krishnaswamy JK Hesse C Untucht C Glage S Behrens G Mayer CT Puttur F Sparwasser T 2012 Absence of Foxp3(+) regulatory T cells during allergen provocation does not exacerbate murine allergic airway inflammation. PLoS One 7:e47102. 10.1371/journal.pone.0047102. 23071726 PMC3468440

[43] Faustino L Fonseca DM Takenaka MC Mirotti L Florsheim EB Guereschi MG Silva JS Basso AS Russo M 2013 Regulatory T cells migrate to airways via CCR4 and attenuate the severity of airway allergic inflammation. J. Immunol. 190:2614–2621. 23390295 10.4049/jimmunol.1202354

[44] Castilow EM Olson MR Varga SM 2007 Understanding respiratory syncytial virus (RSV) vaccine-enhanced disease. Immunol. Res. 39:225–239. 17917067 10.1007/s12026-007-0071-6

[45] Castilow EM Meyerholz DK Varga SM 2008 IL-13 is required for eosinophil entry into the lung during respiratory syncytial virus vaccine-enhanced disease. J. Immunol. 180:2376–2384. 18250447 10.4049/jimmunol.180.4.2376

[46] Walzl G Matthews S Kendall S Gutierrez-Ramos JC Coyle AJ Openshaw PJ Hussell T 2001 Inhibition of T1/ST2 during respiratory syncytial virus infection prevents T helper cell type 2 (Th2)- but not Th1-driven immunopathology. J. Exp. Med. 193:785–792. 11283151 10.1084/jem.193.7.785 PMC2193366

[47] Culley FJ Pollott J Openshaw PJ 2002 Age at first viral infection determines the pattern of T cell-mediated disease during reinfection in adulthood. J. Exp. Med. 196:1381–1386. 12438429 10.1084/jem.20020943 PMC2193991

[48] Krishnamoorthy N Khare A Oriss TB Raundhal M Morse C Yarlagadda M Wenzel SE Moore ML Peebles RS Ray A Ray P 2012 Early infection with respiratory syncytial virus impairs regulatory T cell function and increases susceptibility to allergic asthma. Nat. Med. 18:1525–1530. 22961107 10.1038/nm.2896 PMC3641779

[49] Josefowicz SZ Niec RE Kim HY Treuting P Chinen T Zheng Y Umetsu DT Rudensky AY 2012 Extrathymically generated regulatory T cells control mucosal TH2 inflammation. Nature 482:395–399. 22318520 10.1038/nature10772 PMC3485072

[50] Hussell T Baldwin CJ O'Garra A Openshaw PJ 1997 CD8+ T cells control Th2-driven pathology during pulmonary respiratory syncytial virus infection. Eur. J. Immunol. 27:3341–3349. 9464822 10.1002/eji.1830271233

[51] Srikiatkhachorn A Braciale TJ 1997 Virus-specific CD8+ T lymphocytes downregulate T helper cell type 2 cytokine secretion and pulmonary eosinophilia during experimental murine respiratory syncytial virus infection. J. Exp. Med. 186:421–432. 9236194 10.1084/jem.186.3.421 PMC2198992

[52] Harker JA Lee DC Yamaguchi Y Wang B Bukreyev A Collins PL Tregoning JS Openshaw PJ 2010 Delivery of cytokines by recombinant virus in early life alters the immune response to adult lung infection. J. Virol. 84:5294–5302. 20200251 10.1128/JVI.02503-09 PMC2863826

