# Supplementary material for: Regulatory T Cells Prevent Th2 Immune Responses and Pulmonary Eosinophilia during Respiratory Syncytial Virus Infection in Mice
Source: J Virol. 2013 Oct;87(20):10946–54. doi: 10.1128/JVI.01295-13 (PMC3807299; doi:10.1128/JVI.01295-13)
Supplement: Supplemental material [file supp_87_20_10946__index.html]

Supplemental material 

# Regulatory T Cells Prevent Th2 Immune Responses and Pulmonary Eosinophilia during Respiratory Syncytial Virus Infection in Mice

## Supplemental material

**Files in this Data Supplement:**

- Supplemental file 1 -

  Fig. S1 (Regulatory T cells can be acutely depleted in *Foxpt*DTR mice by using two consecutive diphtheria toxin treatments.)

  Fig. S2 (Expansion of Th2-type regulatory T cells in the inflamed airways of Treg-depleted mice following RSV infection.)

  PDF, 491K
