# Supplementary material for: Regulatory T Cells Prevent Th2 Immune Responses and Pulmonary Eosinophilia during Respiratory Syncytial Virus Infection in Mice
Source: J Virol. 2013 Oct;87(20):10946–54. doi: 10.1128/JVI.01295-13 (PMC3807299; doi:10.1128/JVI.01295-13)
Supplement: Supplemental material [file JVI.01295-13_zjv999098159so1.pdf]

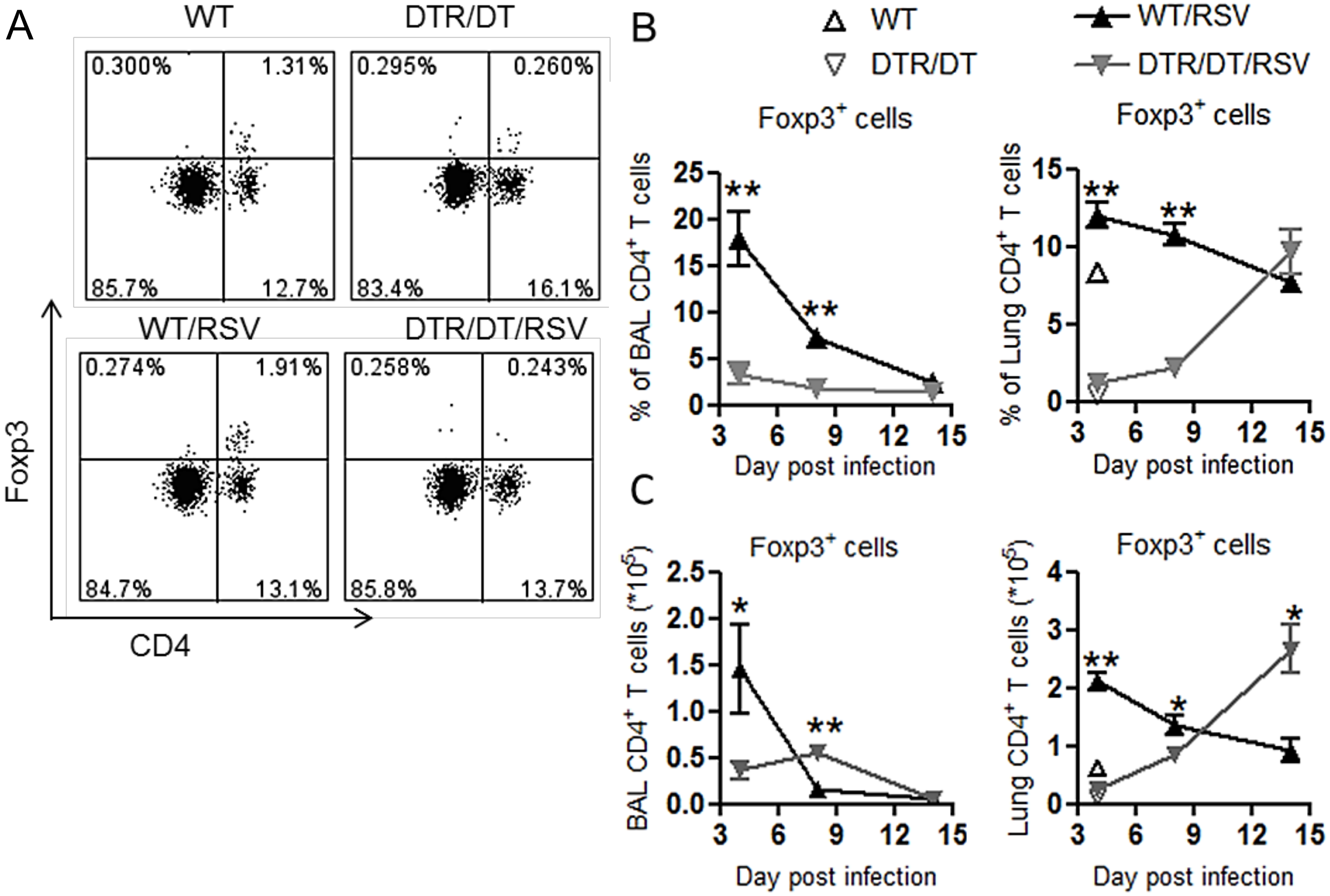

Fig. S1. Regulatory T cells can be acutely depleted in *Foxp3*^{DTR} mice using two consecutive diptheria toxin treatments. Wild type (WT/RSV) or Treg-depleted (DTR/DT/RSV) mice were infected with RSV and diptheria toxin was administered to *Foxp3*^{DTR} mice twice intraperitoneally to deplete Treg cells. Uninfected wild type (WT) or Treg-depleted (DTR/DT) mice were included as controls. A) Representative FACS plots show the percentage of Foxp3^{+} CD4^{+} T cells in the lungs of WT or DTR mice on day 4 post-infection. B) The frequency and C) the total numbers of Foxp3^{+} Treg cells in the airways (BAL) and lungs on Days 4, 8 and 14 post-infection. Data are representative of three independent experiments. * p≤ 0.05, **p≤ 0.01.

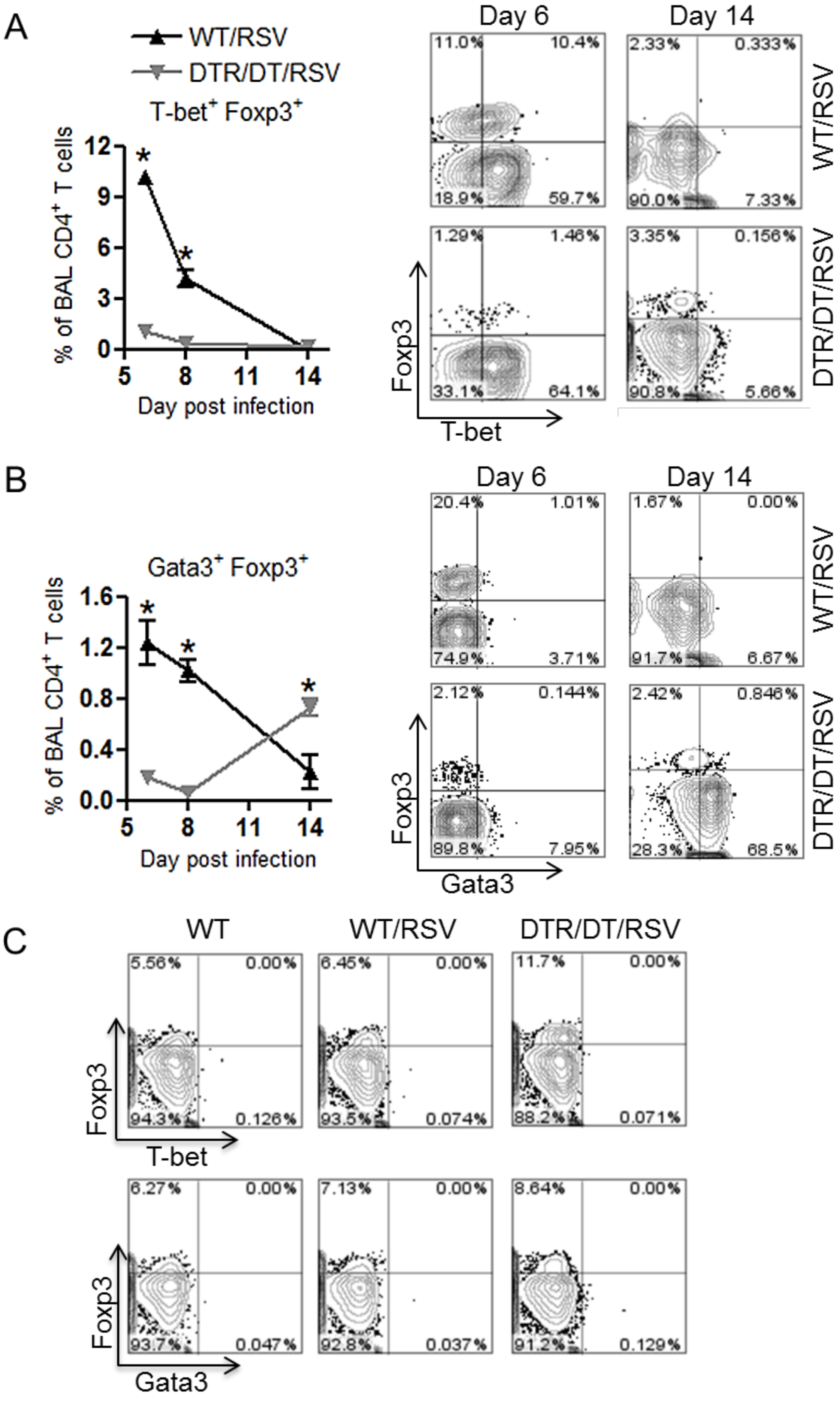

Fig. S2. Expansion of Th2-type regulatory T cells in the inflamed airways of Treg-depleted mice following RSV infection. Wild type (WT/RSV) or Treg-depleted (DTR/DT/RSV) mice were infected with RSV and $CD4^+$ T cells were examined in the tissues using flow cytometry. The frequency of $Foxp3^+$ Treg cells expressing the transcription factorss A) T-bet and B) Gata3 are shown for day 6, 8 and 14 along with representative FACS plots of $CD4^+$ T cells in the BAL on day 6 and 14 post infection. C) Representative FACS plots showing expression of Foxp3, T-bet and Gata3 on CD4+ T cells in the lung on day 14 post infection. Data are representative of two independent experiments (n=3-4 mice per group). * $p<0.05$, ** $p<0.01$.
